# Dissecting racial disparities in multiple myeloma—clues from differential immunoglobulin levels

**DOI:** 10.1038/s41408-020-0314-5

**Published:** 2020-04-28

**Authors:** C. M. Bunce, M. T. Drayson

**Affiliations:** 1 School of Biosciences, University of Birmingham, Birmingham, UK B15 2TT; 2 Institute of Immunology and Immunotherapy, University of Birmingham, Birmingham, UK B15 2TT

**Keywords:** Risk factors, Diseases

Dear Editor,

Catherine Marinac and co-authors recently revisited the longstanding conundrum of racial disparities in the incidence of multiple myeloma (MM), particularly the greater risk of MM in individuals that identify themselves as Black (Blood Cancer Journal (2020) 10:19)^1^. This is an important issue not only in terms of awareness of a population at greater risk of an incurable and devastating blood cancer, but also for the understanding of the molecular and cellular origins, and biology of MM as a whole.

As highlighted by Marinac et al., MM is always preceded by a pre-existing plasma cell dyscrasia termed monoclonal gammopathy of undetermined significance (MGUS)^2,3^. The risk of MGUS in Black populations is also greater than in white populations^4,5^ and is evident at an earlier age^6^. It is therefore possible to surmise that increased susceptibility to MM amongst Black populations relates more to the initiating events that set a B-cell clone on the path to MM occurring more frequently and/or earlier in Black populations, rather than their progression rates being greater. Marinac et al. discuss factors that may underpin this greater risk including socioeconomic factors, genetics and differences in exposure to MGUS and MM risk factors, highlighting the complexity of this issue.

A series of studies spanning three decades from the mid-1960s to the mid-1990s used emerging technologies to measure immunoglobulin levels in populations and how they varied with race, age, gender, life style choices and disease states^7–13^. Collectively these studies identified significantly greater immunoglobulin levels in Black compared with White populations. All studies identified increased IgG in Blacks compared to Whites. One study investigated IgG subtypes and identified increases in IgG1, -2 and -3 but not IgG4^13^. Three studies also identified elevated IgA concentrations in Blacks compared to Whites^9,10^ and in two studies elevated levels of IgG, IgA and IgM were observed^8,9^. These observations raise the possibility that B-cell immunity or activity are elevated in Black compared to White populations and that, by extension, this increased activity may underpin an increased risk of developing MGUS and thereafter MM.

The underlying biology of race differences in Ig levels has been largely unstudied. There is little information available regarding variation in population sizes of the B-cell hierarchy across ethnic groups. However, race differences in response to antigen challenge have been recognised. For example, a study of antibody and B-cell responses to components of the inactivated influenza vaccine (trivalent (IIV3) or quadrivalent (IIV4)), identified higher neutralising IgG responses in African American recipients aged 35–45 compared to age-matched Caucasians^14^. The same study also identified higher baseline numbers of circulating mature naïve, double-negative B cells and antibody secreting cells (ASCs) in African Americans compared Caucasians. A trend towards higher numbers of post vaccination circulating mature naïve, transitional, double-negative and switched memory B cells ASCs in African Americans was also observed^14^. Interestingly, these race-based differences in basal B cell populations and vaccination responses were not seen between older (≥65) African American and Caucasian cohorts^14^.

In separate studies multiparametric flow cytometry-based approaches have been used to measure basal and evoked B cell receptor (BCR) signalling at a single cell level. Although based on small numbers of individuals, B cells from five African Americans had lower anti-IgD induced phosphorylation of multiple BCR pathway components, including the membrane proximal proteins Syk and SFK and components of the PI3K-, MAPK- and NF-κB-pathways than five matched European Americans^15^.

Thus, the differences in MGUS and MM incidence in Black versus White/Caucasian/European populations may not reflect a predisposition for any given B-cell to acquire mutations associated with these disease states, but may reflect a differential overall level of background B-cell immune activity that increases the opportunity for cells to gain such mutations.
